# Electron microscopy study on the transport of lead oxide nanoparticles into brain structures following their subchronic intranasal administration in rats

**DOI:** 10.1038/s41598-022-24018-7

**Published:** 2022-11-14

**Authors:** Marina P. Sutunkova, Ilzira A. Minigalieva, Ivan G. Shelomencev, Larisa I. Privalova, Yuliya V. Ryabova, Anastasiya V. Tazhigulova, Lev A. Amromin, Regina F. Minigalieva, Yuliya M. Sutunkova, Vladimir B. Gurvich, Eugenya V. Makoveeva, Liubov V. Toropova

**Affiliations:** 1 Yekaterinburg Medical Research Center for Prophylaxis and Health Protection in Industrial Workers, 30 Popov Street, Yekaterinburg, Russian Federation 620014; 2 Laboratory of Stochastic Transport of Nanoparticles in Living Systems, Laboratory of Multi‑Scale Mathematical Modeling, Ural Federal University, 51 Lenin Avenue, Yekaterinburg, Russian Federation 620000; 3 Laboratory of Mathematical Modeling of Physical and Chemical Processes in Multiphase Media, Department of Theoretical and Mathematical Physics, Ural Federal University, Lenin Ave., 51, Ekaterinburg, Russian Federation 620000; 4 Otto-Schott-Institut Für Materialforschung, Friedrich-Schiller-Universität-Jena, 07743 Jena, Germany

**Keywords:** Health occupations, Scanning electron microscopy

## Abstract

White outbred female rats were exposed intranasally to 50-µL of suspension of lead oxide nanoparticles (PbO NPs) at a concentration of 0.5 mg/mL thrice a week during six weeks. A control group of rats was administered deionized water in similar volumes and conditions. The developed intoxication was manifested by altered biochemical and cytochemical parameters, as well as behavioral reactions of animals. Using electron microscopy and energy-dispersive X-ray spectroscopy techniques, we revealed deposition of PbO NPs in the olfactory bulb, but not in basal ganglia, and an increase in the number of axons with damage to the myelin sheath in the tissues of olfactory bulb and basal ganglia, changes in the ultrastructure of mitochondria of neurons in the tissues of olfactory bulb and basal ganglia of the brain, and differences in the mitochondrial profile of neurons in different regions of the rat brain. Our results collectively suggest that the central nervous system may be a target of low-level toxicity of lead oxide nanoparticles.

## Introduction

Not only numerically limited occupational cohorts, but also a significant part of the population is exposed to lead due to widespread industrial and anthropogenic processes contributing to lead air pollution, such as non-ferrous metallurgy, coal, diesel and gasoline combustion, battery production, soldering, and tinning. Some of these processes (welding, smelting, etc.) generate metal oxide nanoparticles. Along with submicron particles over 100 nm of a similar composition, they are part of the condensation aerosol polluting the workplace and ambient air (ISO/TR 27628:2007).

There is a growing body of evidence on effects of various air pollutants, including fine particles, on the central nervous system and brain health contributing to an increased risk of stroke, dementia, Parkinson's disease, cognitive dysfunction, as well as neurodevelopmental disorders, depression and other related conditions^1,2^. According to published data, adverse health effects of inhalation exposure to particulate matter may result from two different mechanisms: a direct effect on the brain through the olfactory pathway^3,4^ or an indirect one through an inflammatory response and systemic oxidative stress^5,6^, manifested as a consequence of the local response in the cerebral cortex. These two mechanisms can be both interconnected and act together.

A feature of the impact of metal oxide nanoparticles is their ability to penetrate cell membranes, interact with protein macromolecules, and disrupt organelle functions^7^. Calabró et al.^8^, for instance, demonstrated changes in mitochondrial functions, an increased production of oxidants due to activation of reactive oxygen species, and insufficient protection with antioxidant enzymes in mice exposed to urban air pollution.

The correct transmission of electrical impulses in the CNS is known to occur thanks to the myelin sheath that surrounds axons. Signal protection and isolation, as well as an increase in the rate of transmission of neuronal impulses, are the main functions of this membrane structure^9^. Disruption of the integrity of the myelin sheath leads to errors in signal transmission by axons accounting for various behavioral anomalies^10^. The importance of the role of myelin in the human CNS is emphasized by a wide range of severe neurological disorders, such as leukodystrophy, multiple sclerosis (MS), and peripheral neuropathies, notorious as myelin sheath disorders^7^. Disorganized and damaged myelin can activate microglia, which can fix myelin damage in the body due to the release of inflammatory mediators^11^.

As a result of our literature search for toxicological studies involving intranasal administration of lead oxide nanoparticles (PbO NPs), we have found no publications comparing data on the cellular accumulation of nanoparticles and changes in intracellular morphology in the brain of rats established using electron microscopy with data on behavioral reactions.

### Nanoparticles in the brain

**Figure 1:**
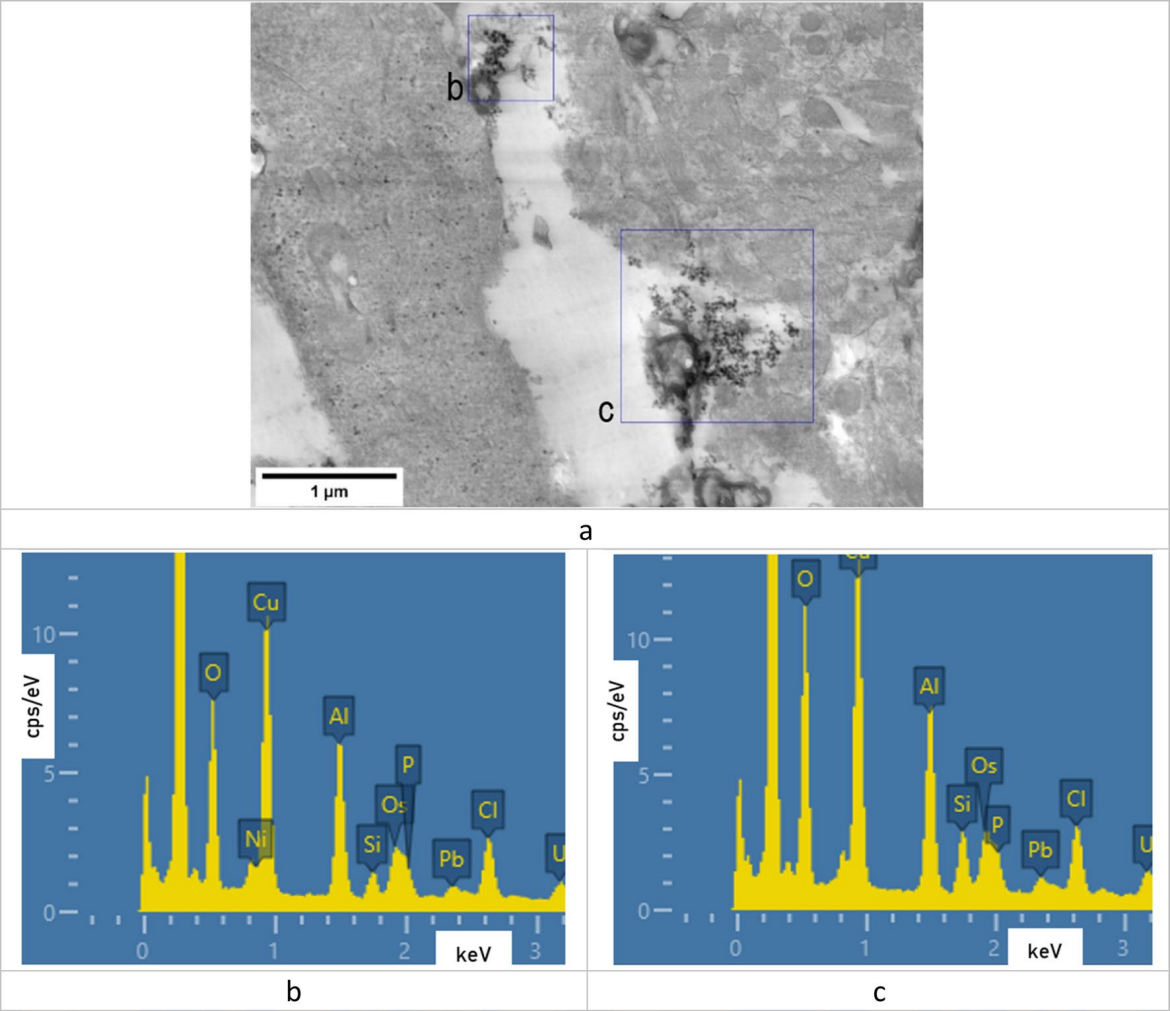
(**a**) STEM image of the olfactory bulb tissues with deposits of lead oxide nanoparticles; (**b**), (**c**) EDS spectra of the regions marked on image (**a**).

Electron microscopy showed deposited lead oxide nanoparticles in olfactory bulb tissues of the brain of rats, their composition confirmed by the EDS detector (Figure 1).

### Myelin sheaths of axons

**Figure 2:**
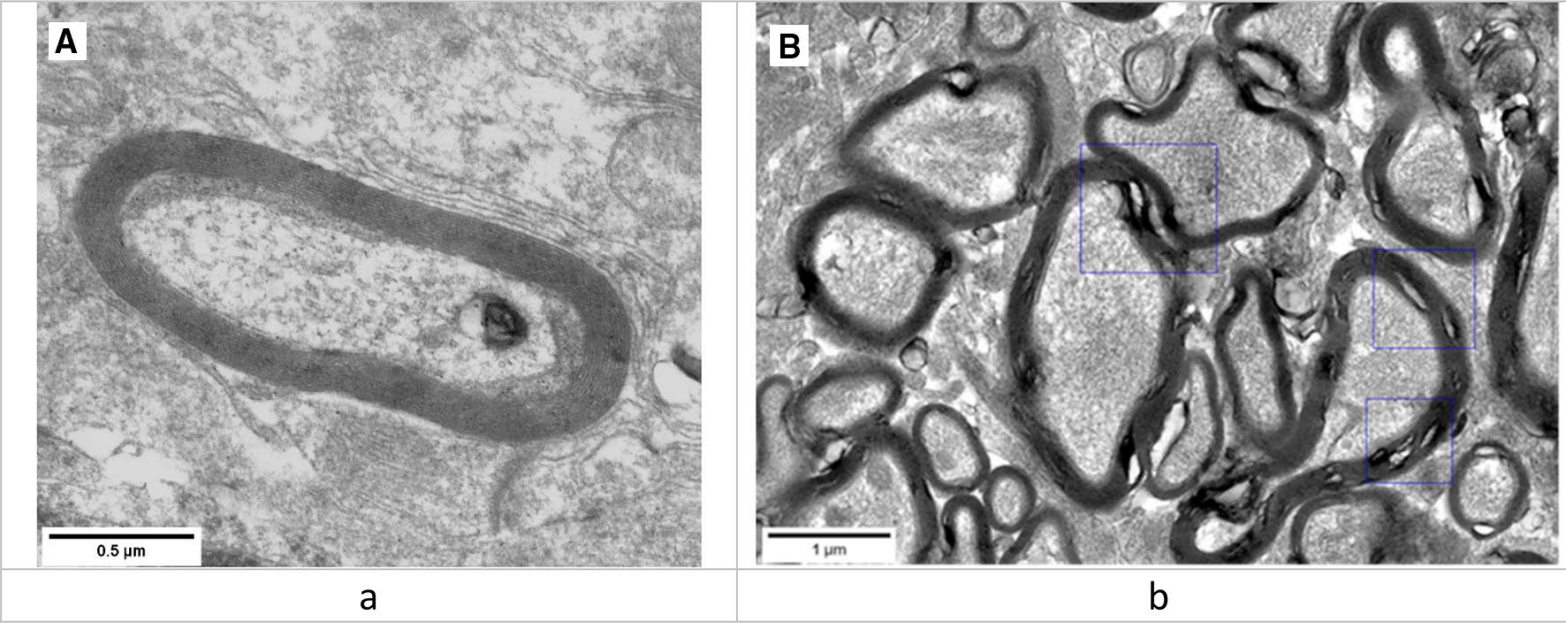
Representative STEM images of normal (**a**) and damaged (**b**) myelin morphotypes.

Morphological changes in myelin sheaths of axons in both parts of the rat brain were studied using electron microscopy. Myelin sheath disorders were represented by multiple craters of different diameters and circumferences (Figure 2).

**Figure 3:**
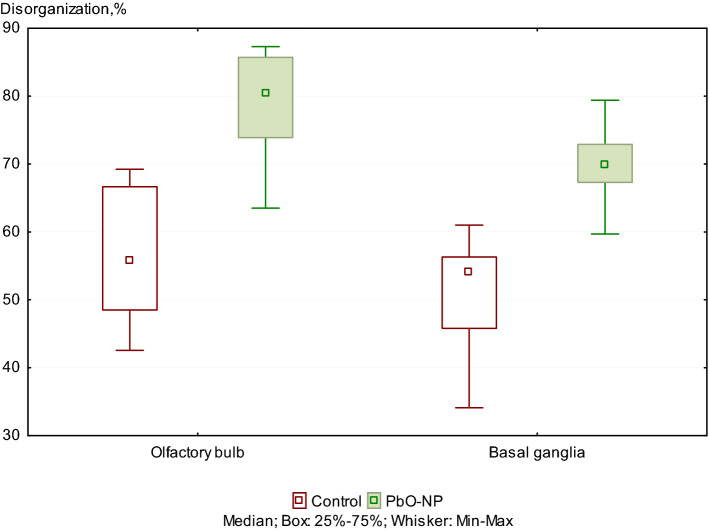
Damages to the ultrastructure of myelin sheaths of axons in the control and PbO NPs exposure groups of rats. Note: Values are given as a fraction of axons with damaged sheaths in the total number of axons detected. Significant differences in damage to the ultrastructure of the myelin sheaths of axons between brain regions in both groups were not found.

The examination of the ultrastructure of the myelin sheath of axons showed a 19% and 21.8% increase in the number axons with the damaged myelin sheath in olfactory bulb and basal ganglia tissues of the exposed animals compared to the controls (Figure 3).

**Figure 4:**
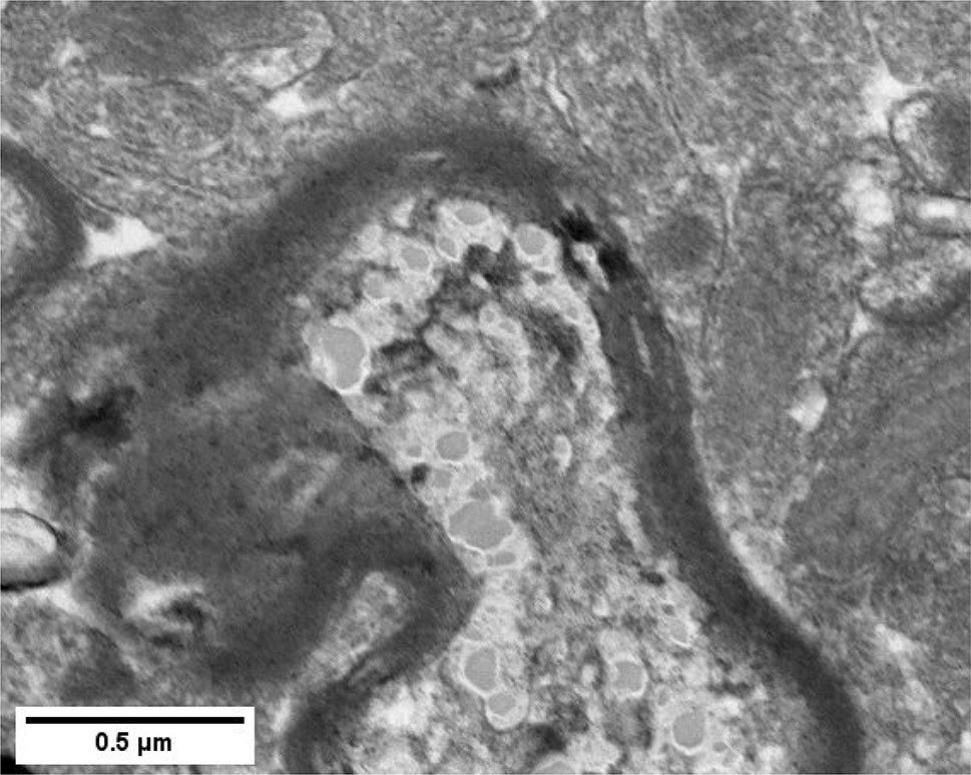
STEM images of damaged axons with loose myelin sheaths in the olfactory bulb of the rats exposed to PbO NPs.

Moreover, the axonal damage was found in both olfactory bulb and basal ganglia in the exposed rats, but not in the controls (Figure 4).

### Neuronal mitochondria

**Figure 5:**
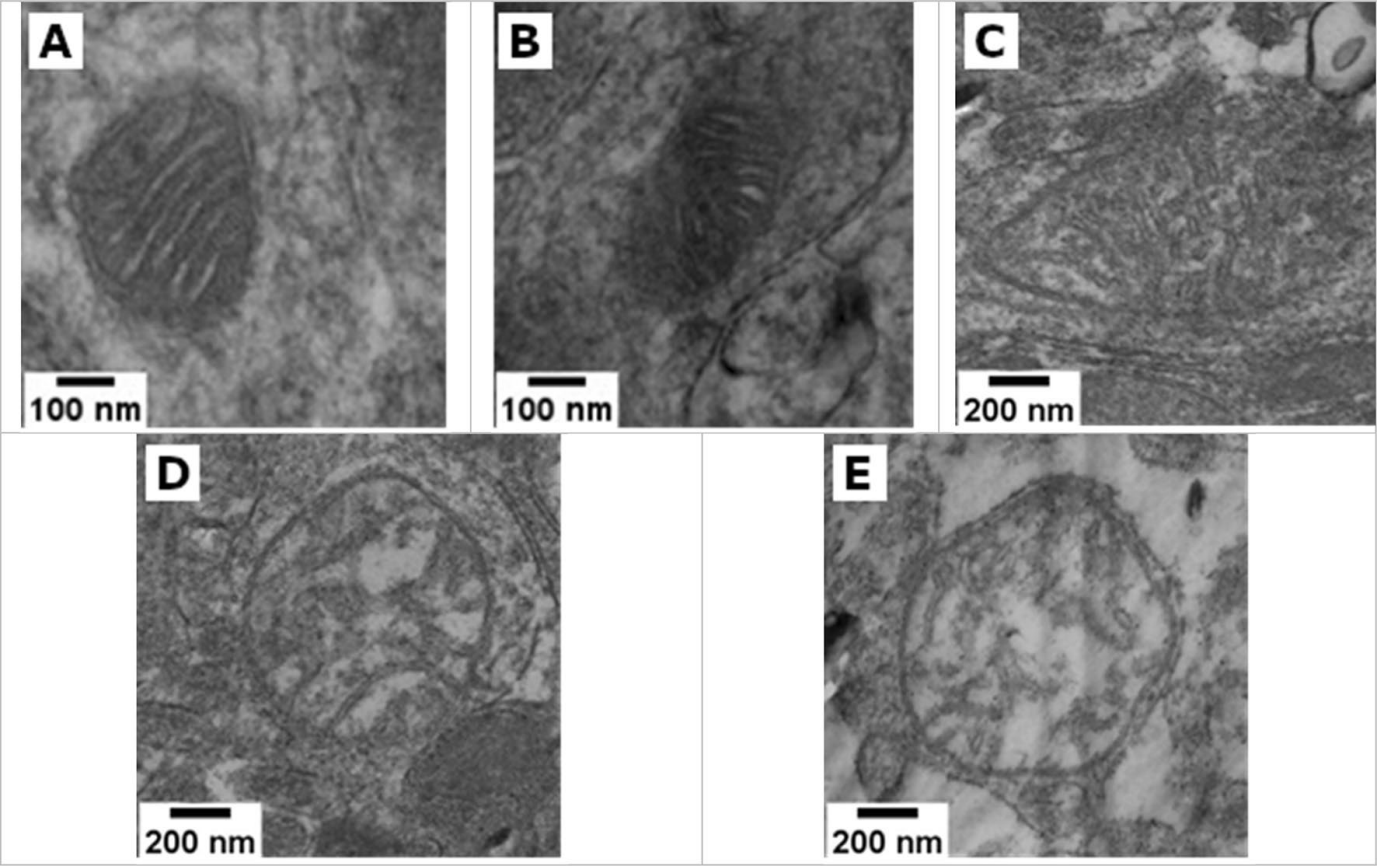
Representative STEM images of normal (**A**), normal vesicular (**B**), vesicular (**C**), swollen vesicular (**D**), and swollen (**E**) mitochondrial morphotypes found in the tissues of both studied groups.

According to the classification suggested by Mei G. Sun^19^, we identified the following consecutive stages of change in the topology of the inner mitochondrial membrane: normal, normal vesicular, vesicular, vesicular swollen, and swollen. Observations made based on the analysis of micrographs of neuronal mitochondria in both groups confirm the presence of these five morphotypes (Figure 5). The images show signs of membrane double contour damage, destruction of the cristae, and clearing of the mitochondrial matrix.

Representative STEM micrographs are shown in Figure 5.

**Figure 6:**
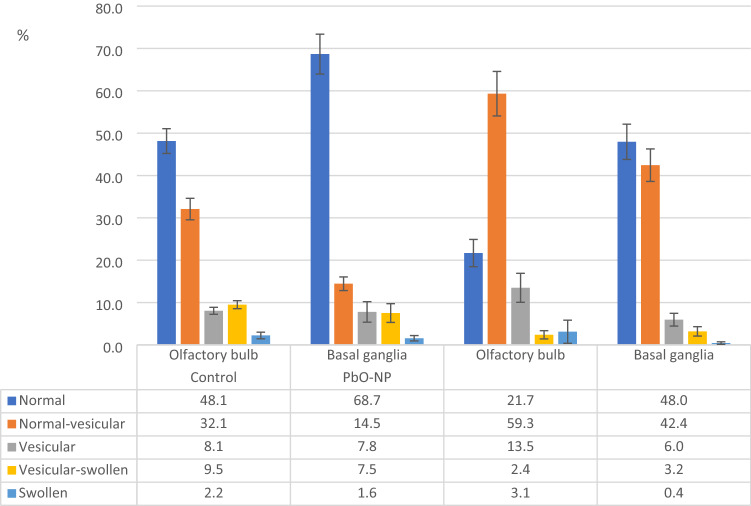
Neuronal mitochondrial ultrastructure damages in the control group and PbO NP exposure groups by brain regions. Notes: Values are given as the arithmetic mean of the fraction of mitochondria in the total number of neuronal mitochondria found; whiskers show the standard error of the mean.

All mitochondria found in the ultrathin section were classified by two independent researchers based on inner membrane topology, and the proportion of each mitochondrial morphotype was estimated (Figure 6).

The two-way ANOVA of collected data proved the differences in the distribution of mitochondrial morphotypes observed and described above, both depending on the group and on the area of the brain (FGroup(4.20)=28.378, *p*<0.001; FTissue(4.20)=13.740, *p*=0.000015). The additional one-way analysis of variance confirmed those differences (Table S1).

In the olfactory bulb tissues of the animals exposed to PbO NPs, we observed a decrease in the percentage of normal and vesicular swollen mitochondrial morphotypes of their total number by 26.4% (*p*=0.003405, U-test M-U) and 7.1% (*p*=0.005341, U-test M-U), respectively, compared with the control group of animals, while the proportion of mitochondria of the normal vesicular morphotype showed an opposite trend increasing by 27.2% following the exposure (*p*=0.003405, U-test M-U). In the tissues of basal ganglia, the pattern of changes in the mitochondrial profile under effect of PbO NPs was preserved only for the mitochondria of the normal and normal vesicular morphotypes. The percentage of normal mitochondria decreased by 20.7% (*p*=0.026810, U-test M-U), while the percentage of normal vesicular mitochondria, on the opposite, increased by 27.9% (*p*=0.003405, U-test M-U). In general, mitochondrial morphotype distribution in the samples of animals exposed to PbO NPs was shifted towards the normal vesicular morphotype whereas in the controls the morphotypes were shifted towards the normal morphotype.

At the same time, the differences in the distribution of mitochondrial morphotypes were also found between different areas of the brain in both groups. In the exposed animals, the percentage of mitochondria of the normal morphotype was 26.3% lower in olfactory bulb samples than in those of basal ganglia (*p*=0.005075, U-test M-U). In the control group samples, the percentages of mitochondria of the normal and normal vesicular morphotypes differed. In basal ganglia samples, the proportion of normal vesicular mitochondria was 20.6% higher (*p*=0.021451, U-test M-U) compared to olfactory bulb samples, while the percentage of normal vesicular mitochondria, on the contrary, was 17.6% lower (*p*=0.002165, U-test M-U). The results of a pairwise analysis using the Student’s t test were similar.

### Condition of experimental animals

**Figure 7:**
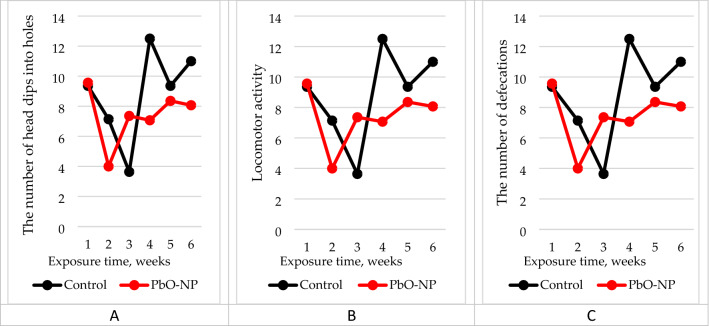
Results of the open field test of the experimental animals: (**A**) the number of head dips into holes; (**B**) locomotor activity; (**C**) the number of defecations. The abscissa shows the exposure time in weeks, the ordinate shows the magnitude of effect.

According to the results of behavioral tests, the number of head dips into holes and locomotor activity of the rats exposed to PbO NPs was 16% and 12% smaller than in the controls, respectively (Figure 7, Table S2). The number of bowel movements during the experiment increased by 28%.

The summation threshold index in the exposed group of rats was increased by 1.5%.

**Figure 8:**
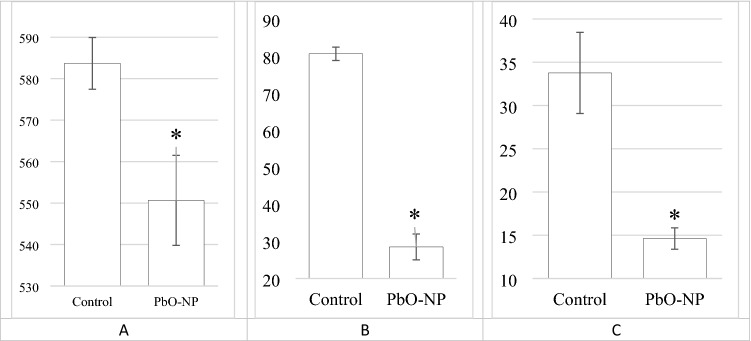
Changes in the activity of succinate dehydrogenase in rat blood lymphocytes (**A**), the concentration of reduced glutathione in blood hemolysate (**B**) and ceruloplasmin in blood serum (**C**) under effect of PbO NPs. The magnitude of the effect is marked on the y axis. *Note* *Statistically different from the control group (*p* < 0.05).

The PbO NP exposure induced an increase in the level of myelin basic protein in blood serum by 34% and a decreased in the brain weight by 5.7% (Table S1). Both the activity of succinate dehydrogenase in blood lymphocytes, the level of reduced glutathione in blood hemolysate and ceruloplasmin in blood serum decreased (Figure 8).

## Discussion

### Detection of nanoparticles in brain tissues

The methods of electron microscopy in conjunction with the use of energy dispersive X-ray spectroscopy enabled to determine localization of nanoparticles in brain tissues. This approach determines a high accuracy of results in contrast to the isolated use of electron microscopy as a visualization source. A similar level of significance is achieved by separating the author’s judgment about the observed objects and replacing it with EDS readings.

The fact that PbO NPs were not found in the basal ganglia samples and their presence in those of the olfactory bulb indicates their uneven distribution in the rat brain (Figure 1). We suppose that PbO NPs were not detected in basal ganglia tissues because they had either failed to penetrate into the deeper regions of the brain in the form of particles, or their number was too small for EM and EDS detection compared with the olfactory bulb. Particle detection could be impeded by a relatively low resolution of the method used. At the same time, this finding is consistent with the theory of nanoparticle migration to the brain along the olfactory nerve following inhalation exposure. This feature has been noted for both PbO and other metal-containing NPs elsewhere^20,21^.

It should be noted that PbO NPs were localized in the cytoplasm of neurons, on the walls of blood vessels, and in the intercellular space, but were not found inside any subcellular structures. Other researchers found nanoparticles in intracellular structures, including mitochondria, and in the nucleus^22,23^. This discrepancy in results can be explained by several factors. First, we used a relatively low concentration of nanoparticles in the suspension, and the cumulative dose was therefore lower than in other studies. In addition, with intranasal administration, part of the suspension bypassing the nasopharynx enters the lungs and gastrointestinal tract, which leads to an even greater decrease in the olfactory exposure. Second, the aforementioned teams used nanoparticles of different chemical nature—silver^22,23^ and gold^23^. Third, the dimension of the particles might have played the principal role: in the studies of Cramer S. et al. the d90 value ranged from 28 to 34 nm, while Panzarini E. et al. reported penetration of 10-nm particles through the GERD barrier. The comparatively large size (49.6 ± 16 nm) of the particles we used might have prevented them from penetrating into the cell.

In the light of the foregoing, we conclude that lead oxide nanoparticles can penetrate brain structures following their intranasal administration in the form of a suspension and distribute unevenly in different parts of the rat brain. This conclusion requires further research to determine the patterns of localization of PbO NPs in the brain and to establish the dependence of PbO NP distribution there on physical characteristics of nanoparticles, the dose and duration of exposure, and the administration technique. At the same time, it is necessary to study correlations between the presence of nanoparticles in tissues, cells, and organelles and their ultrastructural damage.

### Myelin sheaths of axons and changes in animal behavioral responses

The correct transmission of electrical impulses to the CNS is possible due to the myelin sheath that surrounds the axons. Signal protection and isolation, as well as an increase in the speed of transmission of neuronal impulses, are the main functions of this membrane structure. The disruption of the integrity of the myelin sheath leads to errors in signal transmission by axons, which cause various behavioral abnormalities. The importance of the role of myelin in the human CNS is emphasized by a wide range of severe neurological disorders, such as leukodystrophy, multiple sclerosis, and peripheral neuropathies, all of which are united by the fact that myelin sheaths are impaired^24^. Disorganized and damaged myelin can activate microglia, which can fix myelin damage in the body due to the release of inflammatory mediators^11^. The above disorders may result from both natural processes of myelin degradation and exposure to toxic substances^25,26^. In this context, the comparison of the proportions of damaged membranes is of key importance, since the fact of myelin damage was confirmed not only for the PbO NP exposure group, but also for the control group.

In this study, the destruction of the myelin sheath of axons at the ultrastructural level was shown by electron microscopy. The detected changes are represented by degradation of the myelin sheath of axons with an increase in the proportion of the cytoplasm in the damaged area and breach of its integrity. It should be noted that the latter is focal and does not affect the entire length of the studied shells, remaining localized. Based on the results of assessing the state of the ultrastructure of the myelin sheath of axons, we established that the proportion of axons with impaired myelin sheath increased following intranasal administration of suspended PbO NPs to the animals (Figs. 3 and 4).

Demyelination of nerve fibers in the brain of rats exposed to CuO and ZnO nanoparticles has already been demonstrated in previous studies, which, in the opinion of the authors, allows us to indicate a trend towards low-level CNS toxicity common to metal-containing nanoparticles^21^.

Similar myelin sheath disorders were also described elsewhere^7^ following inhalation exposure to lead nanoparticles in the olfactory bulbs of the brain^7^; this study, however, is distinguished by a large number of sheaths studied.

Our findings are confirmed by changes in behavioral reactions of the exposed animals. They are unidirectional despite lacking statistical significance with the controls.

We observed a weakening of exploratory behavior and locomotor activity in the exposed rats compared to the controls. The former also experienced more stress as shown by the increased number of bowel movements (Fig. 7, Table S1). The summation threshold index in the exposed group of rats was increased by 1.5%, which indicates the predominance of inhibitory processes in the central nervous system (Table S1).

An increase in the level of myelin basic protein in blood serum by 34% and a statistically significant decrease in the brain weight by 5.7% following the PbO NP exposure also supports the relationship of the above reactions with the destruction of the myelin sheath of nerve fibers (Table S1). The latter observation is especially interesting because one of the targets of nanoparticles administered intranasally and primarily deposited in the nasal passages is the brain, into which they migrate along the fibers of the olfactory nerve.

We observed similar alterations in exploratory behavior and CNS inhibition in our previous studies of inhalation exposure to lead oxide nanoparticles^7^, which may indicate the interchangeability of techniques in research into nanoparticle health effects.

### Neuronal mitochondria and cytochemical alterations

Mitochondria play a key role in the mechanisms of cellular apoptosis, which is involved in several types of neurodegeneration^27,28^. Since neuronal mitochondria may be the primary and most vulnerable target of toxic metal-containing nanoparticles, our objective was to establish changes in the mitochondrial ultrastructure that occur in vivo in the rat brain following intranasal administration of suspended PbO NPs.

The results showed that the percentage distribution of mitochondria by morphotypes in the tissues of olfactory bulb and basal ganglia differed significantly, which was true for both the exposed and control animals. This may indicate specificity of this indicator for different areas, even within the same organ, and the need to strictly observe the localization of the study area. At the same time, we revealed a significant increase in the fraction of normal vesicular mitochondria and a decrease in that of normal mitochondria in the neurons of the brain of the exposed animals compared to the controls administered deionized water. This trend is typical for all studied areas of the brain.

Defects in the inner membrane of mitochondria lead to disruption of the enzymatic systems located in it. It is known that the only enzyme of the Krebs cycle located in the inner shell of the mitochondrial membrane is succinate dehydrogenase.

In PbO NP exposed rats, the activity of succinate dehydrogenase in blood lymphocytes decreased significantly (Fig. 8A). This confirms disintegration of the mitochondrial membrane observed by electron microscopy in the cell of the organism exposed to PbO NPs. A decrease in the activity of this enzyme may indicate possible mitochondrial malfunctioning of cells.

The altered mitochondrial function is usually associated with increased oxidative stress in cells^29^. Binding to the sulfhydryl groups of glutathione, lead is known to inactivates its ability to recover and participate in the protection of cells from reactive oxygen species as an antioxidant. When considering biochemical parameters, we observed a statistically significant decrease in the level of reduced glutathione in blood hemolysate (Fig. 8B) and ceruloplasmin in blood serum (Fig. 8C), which together can serve as an indicator of impaired cellular redox status and increased oxidative stress in the body.

The value of the reduction potential of glutathione in cells and tissues depends on GSH concentration, and this fact must be taken into account when comparing the reduction potentials in cells and tissues. Given the fact that almost all known CNS diseases are accompanied by an increase in intracellular oxidation processes and an impairment of the antioxidant system, a quantitative assessment of the redox state for the purpose of early diagnosis and treatment of diseases has been very relevant for many years. Currently, such a parameter as the reduction potential of glutathione or the redox potential of glutathione has become popular for the quantitative characterization of the intracellular redox state. Some researchers believe that glutathione is the main cell restorer. The described processes might be therefore related to toxicity of nanoparticles and their ability to stimulate generation of free radicals^5,6^.

Our results may indicate a redox imbalance that has occurred in the exposed rats and made the cerebral cortex tissue more susceptible to oxidative damage.

## Conclusions

The electron microscopy and EDS findings showed that the subchronic intranasal exposure of rats to lead oxide nanoparticles at a total dose of 0.9 mg per rat (approximately 4.2 mg/kg body weight) led to reliable deposition of PbO NPs in the olfactory bulb, an increase in the number of axons with a damaged myelin sheath and changes in the ultrastructure of neuronal mitochondria in those tissues of the rat brain.

An increase in the number of axons with damage to the myelin sheath, together with an increase in the content of myelin basic protein in the blood serum and a tendency to changes in behavioral responses, indicate inhibition of the central nervous system of the exposed animals.

The observed differences in the mitochondrial profile of neurons in the examined parts of the rat brain, changes in the ultrastructure of the mitochondria of brain cells, combined with a decrease in the activity of succinate dehydrogenase in blood lymphocytes, reflecting the intensity of redox processes in the body, may indicate damage to the mitochondrial functions of cells in the exposed animals.

The observed presence of nanoparticles in some parts of the brain, as well as a decrease in the level of reduced glutathione in the blood hemolysate and ceruloplasmin in the blood serum can serve as an indicator of increased oxidative stress in the exposed animals.

Our findings suggest that the central nervous system may be the target of low-level toxicity of lead oxide. Nevertheless, additional studies of PbO NPs distribution patterns in the body and particularly in the brain remain necessary.

## Methods

### Preparation of suspension and physicochemical characteristics of lead oxide nanoparticles

The suspension of lead oxide nanoparticles was prepared at the “Modern Nanotechnologies” Center for Shared Use of the Ural Federal University, Yekaterinburg, Russian Federation, by pulsed laser ablation of thin metal sheet targets of 99.99% pure lead in sterile deionized water as described elsewhere^12^.

**Figure 9:**
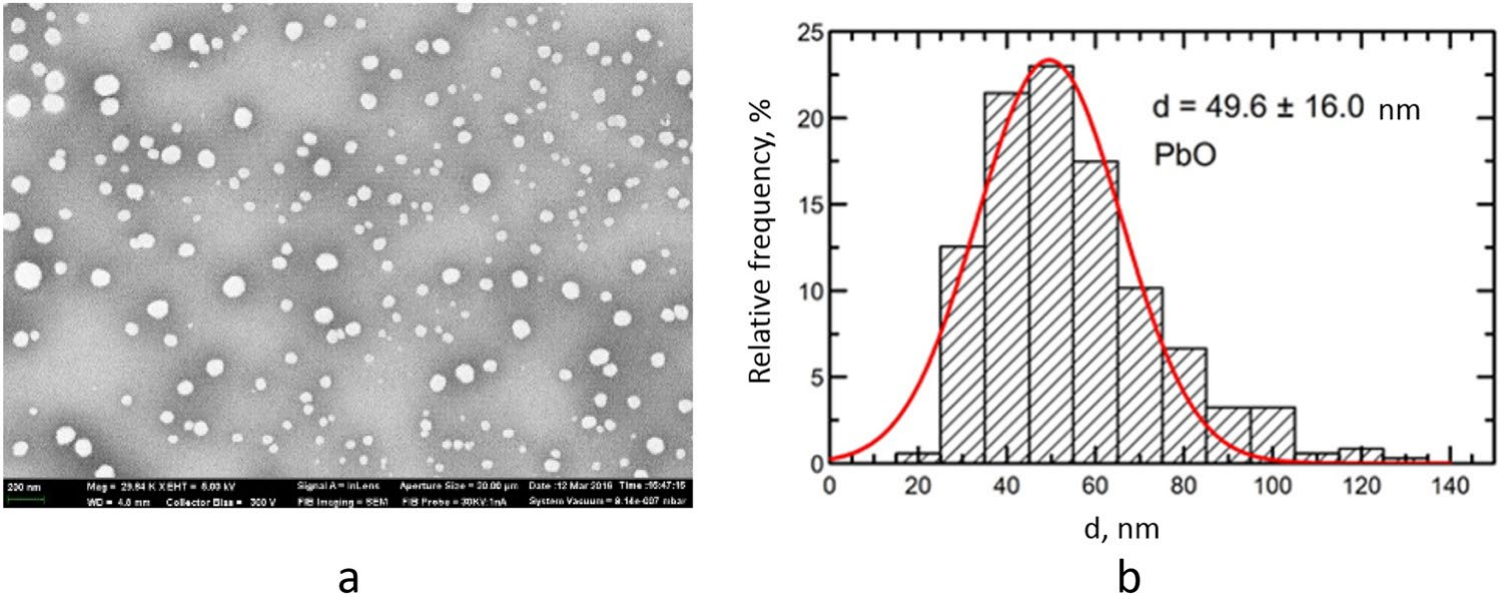
Description of PbO nanoparticles used in the experiment: (**a**) a scanning transmission electron microscopy image of PbO nanoparticles in the suspension at 29,640 × magnification, and (**b**) the function of PbO nanoparticle size distribution.

Scanning electron microscopy (SEM) and the particle size distribution function were used to establish and describe the particle shape and size. The average diameter of the suspended lead oxide nanoparticles equaled 49.6 ± 16 nm (Fig. 9).

The suspension stability was judged by the zeta potential value measured using the Zetasizer Nano ZS size analyzer (Malvern Panalytical, UK) and was high (zeta potential up to 42 mV, *n* = 3, deionized water), which enabled us to increase the particle concentration to 0.5 mg/mL by partial evaporation of water at 50 °C without changing the size and chemical identity of NPs.

### Experimental animals

The experiment was conducted on 3 to 4-month-old outbred female rats of our own breeding with the body weight of ca. 200 g, divided into two groups of 14 animals each. The rats were kept in a specially equipped vivarium room in compliance with the International Guiding Principles for Biomedical Research Involving Animals developed by CIOMS and ICLAS (2012).

50 μL of the suspension of lead oxide nanoparticles at a concentration of 0.5 mg/mL were injected into each nasal passage of fixed experimental animals three times a week for 6 weeks without anesthesia^13^. The total dose for the entire exposure period was 0.9 mg PbO NPs per rat. The control group was administered the same volume of deionized water.

The animals were euthanized by rapid decapitation with immediate blood sampling for biochemical studies. All animals were autopsized and their internal organs, including liver, kidneys, spleen, brain, lungs, and heart, were weighed.

### Exposure assessment

Once a week, the animals were weighed and their behavioral reactions were screened using the open field test^14^, a common measure of exploratory behavior (peeping into holes and sniffing), general locomotor activity, and anxiety, and a summation threshold index^15^. To minimize the stage of adaptation to stress caused by injections, the open field test results and the summation threshold index values were averaged for 6 weeks.

We measured the activity of succinate dehydrogenase (SDH) in blood lymphocytes to assess bioenergetic and redox metabolism. SDH activity was established cytochemically using p-nitroviolet tetrazolium and expressed as the number of formazan granules per 50 cells^16^.

To assess antioxidant mechanisms in the body, we assayed the level of ceruloplasmin in blood serum^17^ and the level of reduced glutathione in blood hemolysate because of importance of its reaction with glutathione in the mechanism of lead transformation^18^. We also detected myelin basic protein in blood serum using the ELISA Kit for Myelin Basic Protein (Cloud-Clone Corp., USA) as recommended by the manufacturer.

### Electron microscopy

**Figure 10:**
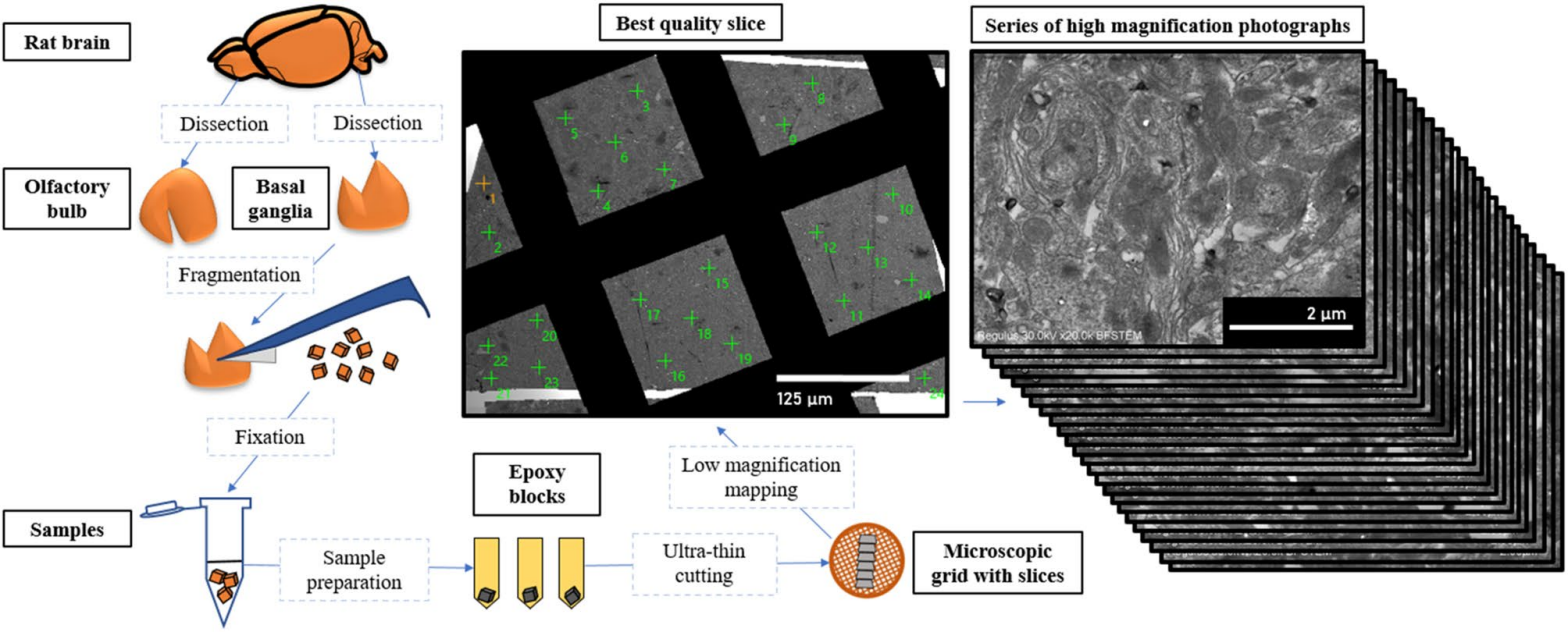
Sampling and sample preparation for electron microscopy.

Randomization of the visual fields and the way of sample preparation for electron microscopy are shown in Fig. 10.

Olfactory bulb and basal ganglia samples from the brains of control (n = 7) and experimental (n = 7) animals were examined by electron microscopy. Samples of the olfactory bulbs and basal ganglia were completely removed from the brain and cut into cubes sized 1 mm^3^. Three to five small pieces of each type of brain tissue were fixed in separate test tubes in a 2.5% solution of glutaraldehyde on 0.1 M phosphate buffer (pH = 7.4). The samples were then post-fixed in a 1% osmium tetroxide solution on 0.1 M phosphate buffer (pH = 7.4), dehydrated in an ascending ethanol series and embedded in Epon812 resin in triplicate. 70-nm-thick sections were cut with a Leica EM UC7 ultramicrotome (Leica Microsystems, Austria) and placed on copper grids. Sections for identification of PbO NPs in brain tissues were stained with a uranyl acetate solution. Sections used to study myelin sheaths and mitochondria were stained with uranyl acetate and lead citrate solutions.

To study morphological changes in myelin sheaths of axons and to assess the state of neuronal mitochondria, three to four series of sections, five to six pieces each, were made for each sample with an interval of at least 100 μm. The minimum of 20 fields of view evenly distributed over the entire surface of the section were examined only in one best-quality ultrathin section from the series using the Hitachi REGULUS SU8220 ultra-high resolution scanning electron microscope in STEM mode. PbO NPs were counted using an Ultim® Extreme Windowless 100 mm^2^ Silicon Drift detector and the spectra were analyzed with using the AZtec software (Oxford Instruments, UK). The images were then processed using ImageJ (National Institutes of Health, US) and GIMP (GNU image manipulation program, Gimp 2.8, developed by GIMP).

To determine the presence of PbO NPs in the rats’ brain tissues, we analyzed 260 brain sections from all animals. To establish the extent of damage to the myelin sheath of axons, we examined samples of the olfactory bulbs and basal nuclei of the brain of all rats. In total, 541 unique regions of the myelin sheaths of rat brain axons were ranked according to the presence or absence of disruptions of the myelin structure.

When estimating the degree of damage to mitochondria following the exposure to PbO NPs, we identified five consecutive stages in the transformation of the inner membrane according to the classification by Mei G. Sun et al.^19^, and ranked 4,320 mitochondria in 156 samples from 13 rats of the control (n = 7) and experimental (n = 6) groups. Morphotyping was carried out by two independent researchers to reduce subjectivity in determining the mitochondrial morphotype, after which the results of the percentage distribution of morphotypes were averaged for each type of tissue and animal.

### Statistical data processing

The statistical significance of data on behavioral reactions and biochemical parameters was assessed using the Student’s t-test (*p* ≤ 0.05). In addition, we compared *p* values estimated by the Student’s t-test and the Mann–Whitney test, and their general coincidence proved the appropriateness of applying the t-test.

The statistical SEM data processing was performed using the Statistica software by StatSoft. The significance of differences between groups was determined using the Student’s t-test, Mann–Whitney U-test, and a one- and two-way ANOVA. The difference between mean values was considered statistically significant if the probability of its random occurrence was below 0.05 (*p* ≤ 0.05).

### Ethics declarations

All methods were carried out in accordance with relevant guidelines and regulations. The manuscript reporting adheres to the ARRIVE guidelines for the reporting of animal experiments. The study was approved by the Ethics Committee of the Yekaterinburg Medical Research Center for Prophylaxis and Health Protection in Industrial Workers (Minutes No. 2 of April 20, 2020).

## Supplementary Information

Supplementary Tables.

## Data Availability

The data presented in this study are available on request from the corresponding author.

## References

[1] Moulton PV, Yang W (2012). Air pollution, oxidative stress, and Alzheimer’s disease. J. Environ. Public Health..

[2] Jayaraj RL, Rodriguez EA, Wang Y, Block ML (2017). Outdoor ambient air pollution and neurodegenerative diseases: The neuroinflammation hypothesis. Curr. Envir. Health Rpt..

[3] Oberdörster G (2004). Translocation of inhaled ultrafine particle to the brain. Inhal. Toxicol..

[4] Sutunkova MP (2018). The most important inferences from the Ekaterinburg nanotoxicology team’s animal experiments assessing adverse health effects of metallic and metal oxide nanoparticles. Toxicol. Rep..

[5] Angelé-Martínez C, Nguyen K, Ameer FS, Anker JN, Brumaghim JL (2017). Reactive oxygen species generation by copper(II) oxide nanoparticles determined by DNA damage assays and EPR spectroscopy. Nanotoxicology.

[6] Paciorek P, Żuberek M, Grzelak A (2020). Products of lipid peroxidation as a factor in the toxic effect of silver nanoparticles. Mater. (Basel).

[7] Sutunkova MP (2020). Manifestation of systemic toxicity in rats after a short-time inhalation of lead oxide nanoparticles. Int. J. Mol. Sci..

[8] Calabró V (2021). Urban air pollution induces alterations in redox metabolism and mitochondrial dysfunction in mice brain cortex. Arch. Biochem. Biophys..

[9] Klocke C (2018). Enhanced cerebellar myelination with concomitant iron elevation and ultrastructural irregularities following prenatal exposure to ambient particulate matter in the mouse. Inhal. Toxicol..

[10] Dąbrowska-Bouta B (2016). Influence of a low dose of silver nanoparticles on cerebral myelin and behavior of adult rats. Toxicology.

[11] Elder A (2006). Translocation of inhaled ultrafine manganese oxide particles to the central nervous system. Environ. Health Perspect..

[12] Privalova LI (2014). Subchronic toxicity of copper oxide nanoparticles and its attenuation with the help of a combination of bioprotectors. Int. J. Mol. Sci..

[13] Katelnikova AE, Kryshen KL, Zueva AA, Makarova MN (2019). Intranasal introduction to laboratory animals. Lab. Anim. Sci..

[14] Guidelines for the use of behavioral reactions of animals in toxicological studies for the purposes of hygienic regulation; (Chisinau, 1980).

[15] Speransky, S.V. *Guidelines for Estimating the Summation Threshold Index for Various Forms of Toxicological Experiment*. (Novosibirsk, 1975).

[16] Narcissov RP (1969). Application of p-nitroviolet tetrazolium for quantitative cytochemistry of human lymphocyte dehydrogenases. Arch. Anat. Histol. Embryol..

[17] Bestuzheva, S. V. & Kolb, V. G. *Clinical Biochemistry.* (Minsk, 1976)

[18] Arkhipova, O. G. *Research Methods in Occupational Health*. (Moscow, 1988)

[19] Sun MG (2007). Correlated three-dimensional light and electron microscopy reveals transformation of mitochondria during apoptosis. Nat. Cell Biol..

[20] Dumková J (2017). Sub-chronic inhalation of lead oxide nanoparticles revealed their broad distribution and tissue-specific subcellular localization in target organs. Part. Fibre Toxicol..

[21] Minigalieva IA (2017). In vivo toxicity of copper oxide, lead oxide and zinc oxide nanoparticles acting in different combinations and its attenuation with a complex of innocuous bio-protectors. Toxicology.

[22] Cramer S (2014). The influence of silver nanoparticles on the blood-brain and the blood cerebrospinal fluid barrier in vitro. J. Nanomed. Nanotechnol..

[23] Panzarini E (2018). Intracellular transport of silver and gold nanoparticles and biological responses: an update. Int. J. Mol. Sci..

[24] Baumann N, Pham-Dinh D (2001). Biology of oligodendrocyte and myelin in the mammalian central nervous system. Physiol. Rev..

[25] Peters A (2009). The effects of normal aging on myelinated nerve fibers in monkey central nervous system. Front. Neuroanat..

[26] Xie F, Liang P, Fu H, Zhang JC, Chen J (2014). Effects of normal aging on myelin sheath ultrastructures in the somatic sensorimotor system of rats. Mol. Med. Rep..

[27] Martin LJ (2010). Mitochondrial and cell death mechanisms in neurodegenerative diseases. Pharm. (Basel).

[28] Savu DI, Moisoi N (2022). Mitochondria – Nucleus communication in neurodegenerative disease. Who talks first, who talks louder. Biochim. Biophys. Acta Bioenerg..

[29] Chew S, Kolosowska N, Saveleva L, Malm T, Kanninen KM (2020). Impairment of mitochondrial function by particulate matter: Implications for the brain. Neurochem. Int..

