# Supplementary material for: Electron microscopy study on the transport of lead oxide nanoparticles into brain structures following their subchronic intranasal administration in rats
Source: Sci Rep. 2022 Nov 14;12:19444. doi: 10.1038/s41598-022-24018-7 (PMC9663722; doi:10.1038/s41598-022-24018-7)
Supplement: Supplementary file 1 — Supplementary Tables. [file 41598_2022_24018_MOESM1_ESM.docx]

Additional information

| Group | Group | Control group | | PbO NP exposure group | |
| --- | --- | --- | --- | --- | --- |
|  | Tissue | Olfactory bulb | Basal ganglia | Olfactory bulb | Basal ganglia |
| Control | Olfactory bulb |  | 10,55* | 25,137* | n/a |
|  | Basal ganglia | 10,55* |  | n/a | 10,35* |
| PbO NP exposure | Olfactory bulb | 25,137* | n/a |  | 5,836* |
|  | Basal ganglia | n/a | 10,35* | 5,836* |  |

**Table S1.** Results of the one-way analysis of variance of the brain area and animal group-dependent percentage distribution of neuronal mitochondria by morphotypes *Notes:* * the values of the Fisher F test statistically different at *p*<0.05; n/a – not analyzed.

| **Parameters** | **Control group** | **PbO NP exposure group** |
| --- | --- | --- |
| *Open field test results:*  Number of head dips into holes | 11.00 ± 1.39 | 8.07±1.29 |
| Locomotor activity | 54.14 ± 3.95 | 45.5±4.78 |
| Number of defecation | 0.50 ± 0.25 | 0.36±0.23 |
| Summation threshold index, s | 11.81 ± 0.85 | 12.85±0.69 |
| Serum concentration of myelin basic protein, ng/mL | 2.44 ± 0.3 | **3.70 ± 0.5 *** |
| Brain weight, g | 1.95 ± 0.03 | **1.84 ± 0.03 *** |
| *Note:* * *p*<0.05 compared with the control group. | | |

**Table S2.** Parameters of the rats’ nervous system following subchronic intranasal exposure to PbO NPs (X̅±Sx).
